# Primary analysis of repeat elements of the Asian seabass (*Lates calcarifer*) transcriptome and genome

**DOI:** 10.3389/fgene.2014.00223

**Published:** 2014-07-25

**Authors:** Inna S. Kuznetsova, Natascha M. Thevasagayam, Prakki S. R. Sridatta, Aleksey S. Komissarov, Jolly M. Saju, Si Y. Ngoh, Junhui Jiang, Xueyan Shen, László Orbán

**Affiliations:** ^1^Reproductive Genomics Group, Strategic Research Program, Temasek Life Sciences Laboratory, The National University of SingaporeSingapore, Republic of Singapore; ^2^Institute of Cytology of the Russian Academy of SciencesSt-Petersburg, Russia; ^3^Theodosius Dobzhansky Center for Genome Bioinformatics, St Petersburg State UniversitySt Petersburg, Russia; ^4^School of Biological Sciences, Nanyang Technological UniversitySingapore, Republic of Singapore; ^5^Agri-Food and Veterinary Authority of SingaporeSingapore, Republic of Singapore; ^6^Department of Animal Sciences and Animal Husbandry, Georgikon Faculty, University of PannoniaKeszthely, Hungary; ^7^Department of Biological Sciences, National University of SingaporeSingapore, Republic of Singapore

**Keywords:** repeated DNA, Asian seabass, transcriptome, rDNA, chromosomes

## Abstract

As part of our Asian seabass genome project, we are generating an inventory of repeat elements in the genome and transcriptome. The karyotype showed a diploid number of 2*n* = 24 chromosomes with a variable number of B-chromosomes. The transcriptome and genome of Asian seabass were searched for repetitive elements with experimental and bioinformatics tools. Six different types of repeats constituting 8–14% of the genome were characterized. Repetitive elements were clustered in the pericentromeric heterochromatin of all chromosomes, but some of them were preferentially accumulated in pretelomeric and pericentromeric regions of several chromosomes pairs and have chromosomes specific arrangement. From the dispersed class of fish-specific non-LTR retrotransposon elements Rex1 and MAUI-like repeats were analyzed. They were wide-spread both in the genome and transcriptome, accumulated on the pericentromeric and peritelomeric areas of all chromosomes. Every analyzed repeat was represented in the Asian seabass transcriptome, some showed differential expression between the gonads. The other group of repeats analyzed belongs to the rRNA multigene family. FISH signal for 5S rDNA was located on a single pair of chromosomes, whereas that for 18S rDNA was found on two pairs. A BAC-derived contig containing rDNA was sequenced and assembled into a scaffold containing incomplete fragments of 18S rDNA. Their assembly and chromosomal position revealed that this part of Asian seabass genome is extremely rich in repeats containing evolutionarily conserved and novel sequences. In summary, transcriptome assemblies and cDNA data are suitable for the identification of repetitive DNA from unknown genomes and for comparative investigation of conserved elements between teleosts and other vertebrates.

## Introduction

Asian seabass (*Lates calcarifer*), also known as barramundi, belongs to the family *Latidae* in the order *Perciformes*, which represents the largest order of vertebrates. Most members of the *Latidae* family are endemic fishes in Africa and the Indian and Pacific Oceans (Luna, 2008). The Asian seabass is a fast-growing, popular food fish found in tropical and sub-tropical fresh and salt waters. Their white flaky meat and the low number of Y-bones have made them a highly rated table fish. This species is a protandrous hermaphrodite: individuals typically mature as males and later reverse their sex to become females (Moore, 1979; Guiguen et al., 1993). The availability of classical aquaculture technologies made the Asian seabass potentially suitable for marker-assisted selection (MAS). Over the past 10 years, together with our two partners in Singapore, we have been working on a MAS project of the Asian seabass. Information encoded in the genes and in the whole genome is required to increase the resolution of selection from MAS to genomic selection in order to make the approach suitable to identify minor-effect genes that control only a small proportion of a trait (Liu and Cordes, 2004; Gjedrem and Baranski, 2009; Gjedrem, 2010).

Recently, we have started the Asian seabass Genome Project in order to develop new platforms for the eventual improvement of the selection process. A substantial part of nuclear genomes of most eukaryotes is occupied by various types of repetitive DNA sequences (Britten and Kohne, 1968) that make the assembly of genomic sequences difficult (Sutton et al., 1995). On the other hand, repeated DNA elements have numerous advantages for genomic studies. They have been extensively applied as physical chromosome markers in comparative studies for the identification of chromosomal rearrangements, the identification of sex chromosomes, chromosome evolution analysis and applied genetics (Ferreira and Martins, 2008).

Repetitive DNA sequences are classified based on genomic organization of repetitive units as dispersed and tandem. Dispersed repeats, represented by various classes of transposable elements encode for proteins which facilitate their replication and integration into nuclear genome (Devine et al., 1997). Tandem repeats are organized in tandem arrays that may be large and consist of thousands or even millions of repetitive units arranged in head-to-tail orientation (Willard, 1991). Chromosome loci rich in polymorphic satellite DNA usually show specific banding patterns and this makes them potentially useful as cytogenetic markers to discriminate individual chromosomes (Lee et al., 2005; Han et al., 2008). The presence of repeat-derived chromosome landmarks enables the identification of individual chromosomes and is a prerequisite to study structural changes accompanying evolution and speciation and to follow chromosome behavior and transmission in interspecific hybrids (Lee et al., 2005).

The processes by which satellites arise and evolve are not well understood; unequal crossing over, gene conversion, transposition and formation of extra chromosomal circular DNA were all implicated (Carone et al., 2009; Enukashvily and Ponomartsev, 2013). The Nucleus Organizer Regions (NOR) are described as highly repetitive genome sites related to the rRNA synthesis. These regions present small, active transcription sites (highly conserved during evolution) and non-transcribed spacing segments (highly variable), organized as two distinct multigene families 47S and 5S rRNA genes. Tandemly arrayed rDNA repeats are composed of hundreds to thousands copies, disturbed by dispersed elements and usually belong to separate chromosomes (Cioffi et al., 2011).

Repeat regions can cause serious problems during genome assemblies, especially for those that involve short reads produced by next generation sequencing (NGS) technologies. This is due to the fact that a fragment from a repeat region can have false overlaps with fragments from other repeat regions, resulting in the merging of unrelated regions and incorrect final assemblies. The fragments from repeat regions are identified by the number of potential overlaps each fragment has based on pairwise comparisons (Sutton et al., 1995).

In this study, we focus on the primary analysis of repeat elements of the Asian seabass transcriptome and genome using approaches based on classical cytogenetics, molecular biology and next generation sequencing technology. Through this work we have demonstrated that the transcriptome assembly and cDNA data contains repeats and it is suitable for the identification of repeat DNAs from an unknown genome and for comparative investigation of conserved repeat elements between species. Altogether thirteen repeat elements, including 5S rDNA and 18s rDNA, have been identified from the Asian seabass genome, together they constitute 8–14% of the genome. All of them produce short transcripts. Some repeats are enriched on specific chromosomes, while others are generally distributed on the karyotype, except for B-chromosomes.

## Materials and methods

### Primary culture and chromosomes preparation

Asian seabass larvae of 1–2 days post-hatching (dph) age from the Marine Aquaculture Center (St John's Island, Singapore) were sacrificed by placing on ice and they were seeded into cell culture dishes in L15 or RPMI medium supplemented with 20% fetal calf serum and antibiotic and antimitotic solution (Sigma). The primary culture was incubated at 29°C with 5% CO. After 1–2 weeks, the cells were incubated with 0.01% of colchicine for 5–6 h. Chromosome spreads were prepared using the method outlined by Pradeep et al. (2011). Chromomycin A3 (CMA3) and DAPI fluorochrome stainings followed the methodologies of Schmid (1980) and Schweizer (1980), respectively.

### DNA and RNA extraction

Tissue samples from brain and gonad of four female and four male individuals were collected and stored at −80°C until use. Genomic DNA (gDNA) was extracted from 1 to 2 dph larvae, as well as the liver, ovary and testis of 5 years old male and female individuals using the standard phenol-chloroform procedure (Sambrook and Russell, 2001). Total RNA was isolated using the Trizol kit (Invitrogen).

### Quantitative real-time PCR (qPCR) analyses

One mg gDNA-free total RNA from the ovary, testis as well as female and male brain, respectively, of adult Asian seabass individuals was reverse-transcribed using a cDNA Synthesis Kit (Qiagene) according to the manufacturer's recommendations. The cDNA samples obtained were diluted 1:10 with sterile water before their use as templates in real-time quantitative PCR (qPCR). Levels of mRNA were determined using qPCR and SYBR Green chemistry on a Stratagene Mx3000P (Agilent Technologies, USA) using elongation factor 1-alpha and ribosomal protein L8 as reference genes. The Relative Expression Software Tool was used to calculate the relative expression of target mRNA. Results are reported as mean ± standard error. Significant differences between means were analyzed with the pairwise fixed reallocation randomization test (Pfaffl et al., 2002). In all cases, a value of *p* < 0.5 was used to indicate significant differences. As the expression levels between the male and female brains were similar their values were combined.

### S1-nuclease treatment

The procedure was modified from the protocol used to isolate Cot-1 DNA (Zwick et al., 1991; Ferreira and Martins, 2008). DNA samples (300 μL of 100–500 ng/μL genomic DNA in 0.5 M NaCl) were boiled for 30 min and the size range of fragmented DNA was checked by electrophoresis in a 1% agarose gel (0.1–5 kb). Samples of 50 μl of fragmented DNA were then denatured at 95°C for 10 min, placed into ice-cold solution for 10 s, and transferred to a 65°C water bath for re-annealing. Following 1 min of re-annealing, the samples were incubated at 37°C for 20 min with 0.5 U of S1 nuclease to permit digestion of single-stranded sequences.

### PCR-based isolation of repetitive DNAs using specific primers

The sequences of Rex1, 5S, and 18S rDNA were amplified directly from total gDNA by specific primers (Supplementary Table S1). Partial sequences of the MoSat_SB, GGSat_SB, and YRep_SB repeat DNAs were detected in the Asian seabass transcriptome dataset. They were PCR-amplified from total gDNA with specific primers (Supplementary Table S1). Primers were designed with PRIMER3 (http://www-genome.wi.mit.edu/genome_software/other/primer3.html) software using the transcriptome and Repbase data as reference sequences. The telomere probe was obtained by PCR-amplification with (TTAGG)_5_ primer.

### Selection of 18S rDNA-containing clones from the BAC library, their sequencing and assembly

The Asian seabass BAC library was a replica of that described earlier (Xia et al., 2010), except this version contained only 38,400 of the 49,152 clones reported earlier. With an average insert size of 98 kb (range: 45–200 kb), this provided a 5.4-fold coverage for the haploid genome. From the library described above, clones from one hundred 384-well plates were processed by a 3D pooling method yielding 140 pooled samples. 5S and 18S rDNA primers (Supplementary Table S1) were used for screening the above pools to identify clones containing 18S rDNA.

### BAC sequencing and assembly

BAC-end sequences were generated by Sanger sequencing using the identical BAC DNA preparations as templates with the universal primers T7 (5′-TAATACGACTCACTATAGGG-3′) and plBRP (5′-CTCGTATGTTGTGTGGAATTGTGAGCC-3′). The inserts of the two BAC clones containing 18S rDNA were sequenced both by Ion Torrent (2,584,864 reads; AIT Biotech, Singapore) and 150 bp paired-end sequencing on Illumina HiSeq (2,584,864 reads; Yourgene, Taipei). The raw reads were corrected with Quake program with parameter *k* = 13 [http://genomebiology.com/2010/11/11/R116/abstract]. Corrected reads from the two NGS platforms were mapped separately with Bowtie program (Langmead et al., 2009) to the human reference genome, to E. coli genome, to the vector sequence (pCC1BA), European seabass (*Dicentrarchus labrax*; http://www.ncbi.nlm.nih.gov/assembly/GCA_000180815.1/); an African cichlid *(Astatotilapia burtoni* http://www.ncbi.nlm.nih.gov/assembly/GCA_000239415.1/, zebrafish (*Danio rerio*; http://www.ncbi.nlm.nih.gov/assembly/GCF_000002035.4/), Nile tilapia (*Oreochromis niloticus*; http://www.ncbi.nlm.nih.gov/assembly/GCA_000188235.2/, Atlantic salmon *(Salmo salar*; http://www.ncbi.nlm.nih.gov/assembly/GCA_000233375.1/) and to the expected rDNA sequences. Reads that mapped only to E. coli, vector, and/or to the human genome were discarded. The remaining reads were assembled into contigs with SPAdes 2.4 assembler with default parameters (Bankevich et al., 2012). Short contigs (<200 bp) with a coverage less than 100x were discarded. The assembled contigs were compared against the nucleotide collection (nr/nt) database with BLAST on NCBI website. To assemble the scaffold drafts, two datasets were used: (1) contigs obtained by single read sequencing (Ion Torrent); and (2) contigs generated from paired-end reads (Illumina HiSeq 2000). The HiSeq-derived data was used for contig assembly, whereas the Ion Torrent-derived data was used for contig positioning according to their physical map relative to HiSeq data (Supplementary Figure S1). For the overlapping Ion Torrent datasets we computed k-mers with the Jellyfish program (Marcais and Kingsford, 2011) that were mapped to assembled contigs with the Bowtie software (http://www.bowtie-bio.sourceforge.net/index.shtml). According to the computed coverage, the Ion Torrent-derived contigs were separated into three groups: those located in the left part of physical map, those located in the middle part (overlap), and the rest located in the right part. Assembled contigs have been deposited at GenBank, under the accession numbers of KF432408–KF432412.

### The partial asian seabass transcriptome assembly

The sequences used for the bioinformatic identification of repeats were obtained from the Asian seabass transcriptome assembly (Supplementary Figure S1). The brief description of the sequencing and assembly of the first batch of HiSeq data is described below. Pooled RNA samples from various organs and developmental stages of Asian seabass were sequenced on Illumina HiSeq 2000 with 2 × 100 pair-end reads. Following quality- and adapter-trimming, a total of ~487 million reads were obtained and deposited to NCBI SRA [SRP033113]. Potentially contaminant reads were removed from the dataset if they did not show any homology with seabass or zebrafish mRNA sequences, resulting in ~485 million clean reads (~242 million pairs) which were then assembled using Trinity (version R2012-06-08) to generate 459,979 contigs.

All these contigs were further assembled using CAP3, resulting in 363,785 contigs. This Transcriptome Shotgun Assembly project has been deposited at GenBank, under the accession number of GAQL00000000 (the version described in this paper, is the first version, GAQL01000000). Contigs with an average read coverage ≥3 were further clustered using CD-HIT-EST resulting in 182,842 contigs.

### Sequence analysis

All sequence comparisons between large sets of sequences were performed using standard algorithms such as BLAST (Altschul et al., 1990). To check for the presence of repeat elements the sequence sets were searched against the Repbase database (Kohany et al., 2006) using CENSOR (Ver: 4.2.28) with follow parameters: default, nofilter, minsim 0.75, show_simple, bprg blastn, mode norm and the Nile tilapia repeat collection, http://cowry.agri.huji.ac.il/DATA_SET_RM/RepeatLib.html (Shirak et al., 2009). Phylogenetic relations between species have been determined by Interactive Tree Of Life software v2.2.2. (Letunic and Bork, 2011). Comparative transcriptome sequence analysis with Repbase of different species is presented in Supplementary Table S3. For detection of microsatellites, the Tandem Repeat Finder version 2.02 software (Benson, 1999) was used with the following parameters: match: 2, mismatch: 7, delta: 7, PM: 80, PI: 10, minscore: 50, maxperiod: 300 (Supplementary Table S4).

### cDNA libraries

The following five additional cDNA sequence datasets were used in the comparative analysis: Nile tilapia (*Oreochromis niloticus*; 67 Mb), three-spined stickleback (*Gasterosteus aculeatus*; 45 Mb), Japanese medaka (*Oryzias latipes*; 38 Mb), mouse (*Mus musculus*; 159 Mb) and human (*Homo sapiens*; 297 Mb). These datasets were obtained from the Ensembl genome browser (http://www.ensembl.org/index.html).

### Cloning and sequencing

The restricted DNA fragments and PCR products were cloned into pGEM-T plasmid vector (Promega) and transfected in DH5a E. coli competent cells according to standard protocols (Sambrook and Russell, 2001). Positive recombinant clones were sequenced by Sanger protocol.

### Southern and dot-blot hybridization

Genomic DNA was dot-blotted onto positively charged nylon membranes (Hybond-N++, Amersham) in a series of dilutions ranging from 50 ng to 2 μg. Filter-immobilized DNA was hybridized with PCR products of repeat DNA labeled with dig-dUTP. The plasmid and PCR product with a cloned fragment was used to produce the calibration curve which was used to estimate the proportion of the sequences in the genome. Hybridization was performed at 42°C in a solution composed of 6xSSC, 0.5% SDS, 5xDenhardt, 50 μg/ml salmon sperm, 50% formamide, 0.01 mol/L EDTA, and 20 ng/ml probe (Cardinali et al., 2000; Sambrook and Russell, 2001). Densitometric quantification from the dot-blots was performed using Gel-Pro Analyzer Version 3.1.00.00.

For Southern hybridization, samples of genomic and BAC DNA (10 μg) were completely or partially digested with various restriction enzymes (Hind III, RsaII, EcoRI, BamHI, or HinfI). The digestion products were subjected to gel electrophoresis on 1–1.5% agarose gels and southern-transferred to a Hybond-N++ nylon membrane. The hybridized DIG-labeled probe was detected with an antidigoxigenin alkaline phosphatase-conjugated antibody (Roche, Germany) according to the manufacturer's protocols.

### Karyotypes and fluorescent *in situ* hybridization (FISH)

In order to determine the karyotype of the Asian seabass, 20 good metaphase plates were used. The classification of chromosomes followed (Levan et al., 1964). Metacentrics (M) and submetacentrics (SM) were described as two-arm chromosomes, whereas subtelocentrics (ST) and acrocentrics (A) as one-arm chromosomes.

All probes were labeled with digoxigenin-11-dUTP (DIG) and biotin-16-dUTP (Roche) for FISH. The labeled nucleotides were incorporated into fragments by PCR, using M13 forward and reverse primers. The slides were denatured in 70% formamide/2xSSC for 3 min at 72°C. For each slide, 50 μl of hybridization solution (containing 1 μg of each labeled probe, 50% of formamide, 2x SSC, 10% dextran sulfate), was denatured for 10 min at 75°C and allowed to prehybridize for 1 h at 37°C. Hybridization took place for 16–18 h at 37°C. Post-hybridization washes were in 4x SSC for 5 min at 73°C and 2x SSC for 5 min at room temperature. Following a wash in PBST (PBS, 0.1% Tween), slides were incubated with a Rodamin Red-avidin (Invitrogen) and FITC-conjugated anti-DIG antibody (Roche). Finally, the slides were counterstained with DAPI and mounted in an antifade solution (Vectashield, Vector laboratories, Burlingame, CA, USA). Images were captured with a Nikon (CCD) camera on a Zeiss/MetaMorph epifluorescence microscope and were optimized using Adobe Photoshop CS2.

## Results

### The karyotype of asian seabass showed 24 pairs of autosomes and a variable number of B-chromosomes

**Figure 1:**
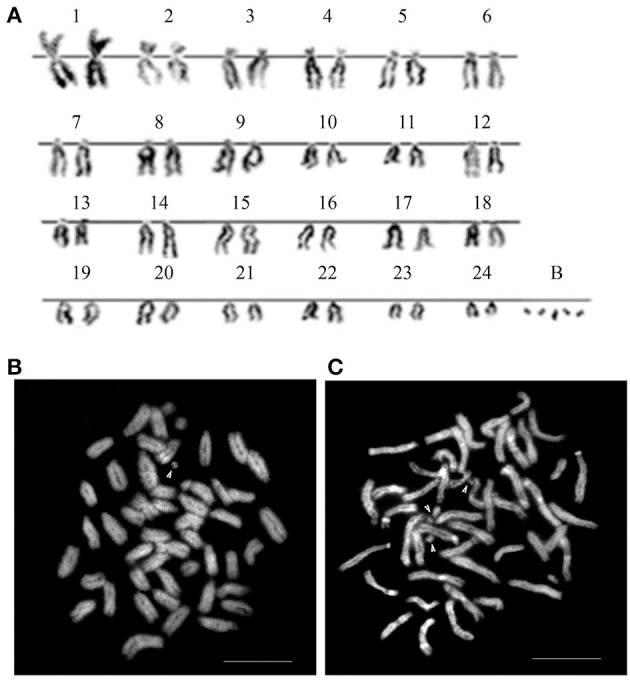
**The karyotype of Asian seabass contains 24 pairs of A-chromosomes and a variable number (2–10) of additional B-chromosomes. (A)** The karyotype. Metaphase spreads stained with DAPI **(B)** and Chromomycin A3 **(C)**. Arrowheads in **(B)** indicate AT-rich B-chromosomes, whereas those in **(C)** label GC-rich ones.

The karyotype of Asian seabass embryos showed a diploid number of 2*n* = 48 and a karyotype formula of 1M + 1SM + 11ST + 11A (*FN* = 37). The individual karyotypes also contained a variable number (2–10) of additional microchromosomes, suggesting the occurrence of B-chromosomes (Figures 1A,B). When stained with CMA3, most of chromosomes showed the presence of a GC-rich block near the pericentromeric (periCEN) chromatin (Figure 1C). All chromosome spreads had at least two GC-rich B-chromosomes (Figure 1C), whereas AT-rich B-chromosomes (Figure 1B) were detected only in 60% of the chromosome spreads.

### Isolation, cloning and chromosomal localization of repetitive elements from the asian seabass transcriptome and genome

**Figure 2:**
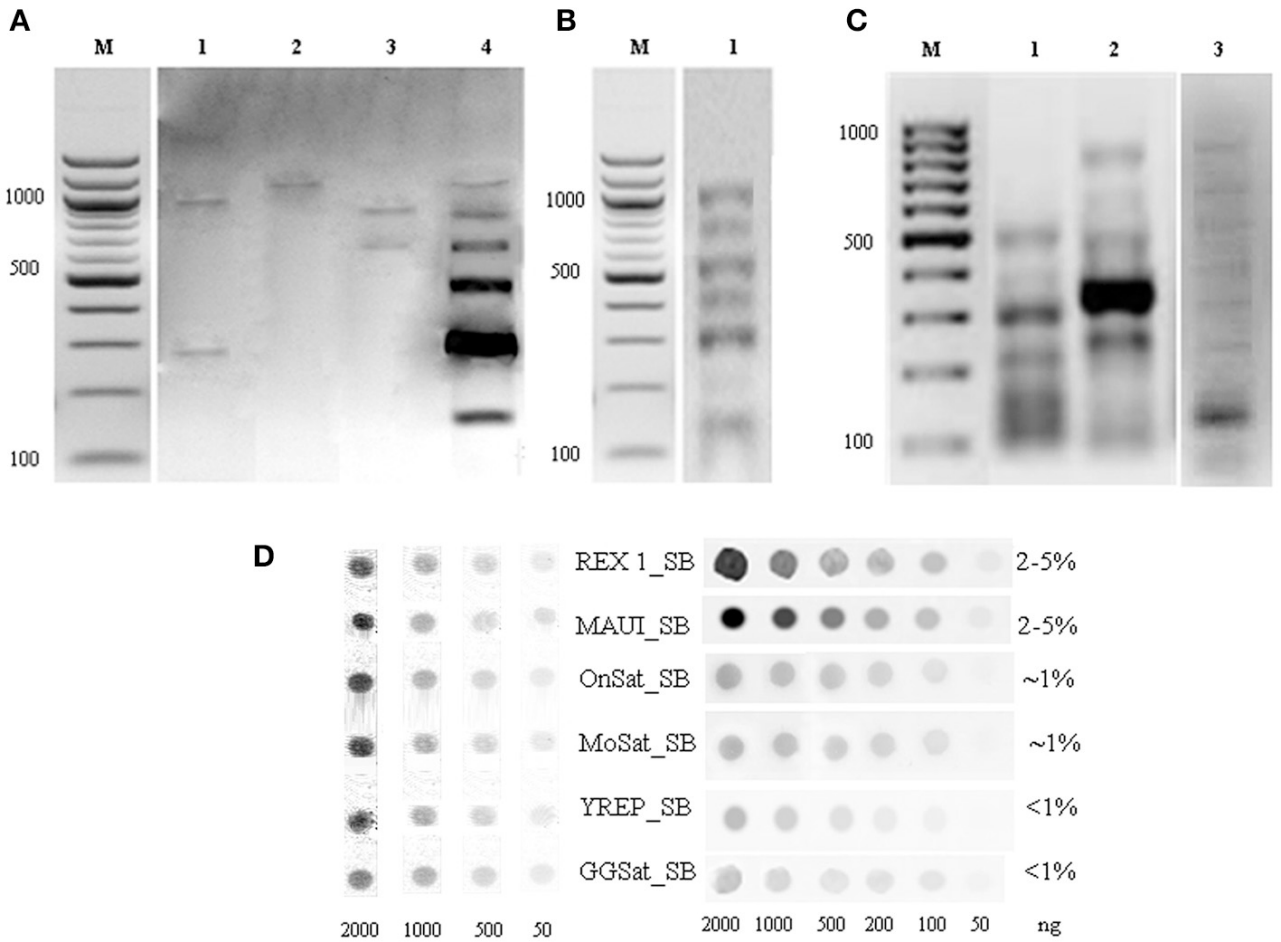
**Southern blot hybridization of Asian seabass genomic DNA (gDNA) digested by restriction endonucleases and hybridized with repeat-derived probes. (A)** gDNA digested by EcoRI (1), HapI (2), BamHI (3), or RsaI (4) and hybridized with an OnSat_SB probe. **(B)** gDNA digested by HinfI and hybridized with a MoSat_SB (1) probe. **(C)** PCR fragment distribution of GGSat_SB (1), YRep_SB (2), and MoSat_SB (3) amplified with sequence-specific primers indicated in Supplementary Table S1. Probes were labeled with DIG-dUTP. M—molecular mass marker of 100 bp, the corresponding molecular masses are indicated on the left. **(D)** Dot-blot hybridization of Asian seabass genomic DNA with DIG-labeled probes for six different repeats allowed for the estimation of their proportion in the genome. Genomic repeat content (%) is indicated in the right column. Samples used for the DNA standard are at the left side.

We have combined the power of three different approaches for the isolation and characterization of repetitive elements from the Asian seabass genome (Supplementary Figure S1). The first approach was digestion of genomic DNA by S1-nuclease. Out of the 12 sequences cloned, nine were unknown short sequences of 100–300 bp length and not utilized for subsequent analysis. Two of the remaining three have shown similarity with OnSatB (GenBank ID:S57288) periCEN DNA of the Nile tilapia genome (Supplementary Table S1) and they were called OnSat_SB (Table 1). The divergence between the two OnSat_SB sequences was less than 1%. Southern blot hybridization of total DNA with OnSat_SB probe yielded satellite-like character ladders with steps of 200 bp length (Figure 2A). The third sequence was a short fragment of a CR1 non-long-terminal-repeat (non-LTR) retrotransposon MAUI_SB (Table 1), which showed similarity with the ORF1 part of a non-LTR retrotransposon, called MAUI from the green spotted pufferfish (*Takifugu rubripes*) genome (Poulter et al., 1999).

**Table 1:** **The inventory and characterization of nine repeats isolated from the Asian seabass transcriptome and genome**.

**Repeat name**	**NCBI access. No**	**Size of clones (bp)**	**AT%**	**Repeat description**		
				**Type**	**Location**	**Class of repeats**
MAUI_SB	KC842206	164		CR1, non-LTR retrotransposon,	periCEN^*^, dispersed	Dispersed
Rex_SB	KC842207	551	48	CR1, non-LTR retrotransposon	periCEN	
	KC842208	560				
YREP_SB	KC842210	326	73	unknown	periCEN, dispersed	
GGSAT_SB	KC842209	302	66	unknown	dispersed	
OnSat_SB	KC842204	233	56	satellite DNA	periCEN	Tandem
	KC842205	217				
MoSat_SB	KC842211	148	59	satellite DNA	periCEN	
	KC842212	155				
Telomere (TTAAGGG)5	N/A^**^	35	50	Tandem repeat	Telomere	
5S rDNA	KC842203	556	ND^*^	Ribosomal DNA	periCEN	
18S rDNA	KC842201	306	ND^*^	Ribosomal DNA	periCEN	
	KC842202	756				

* periCEN, pericentromeric.

** N/A, not applicable.

**Figure 3:**
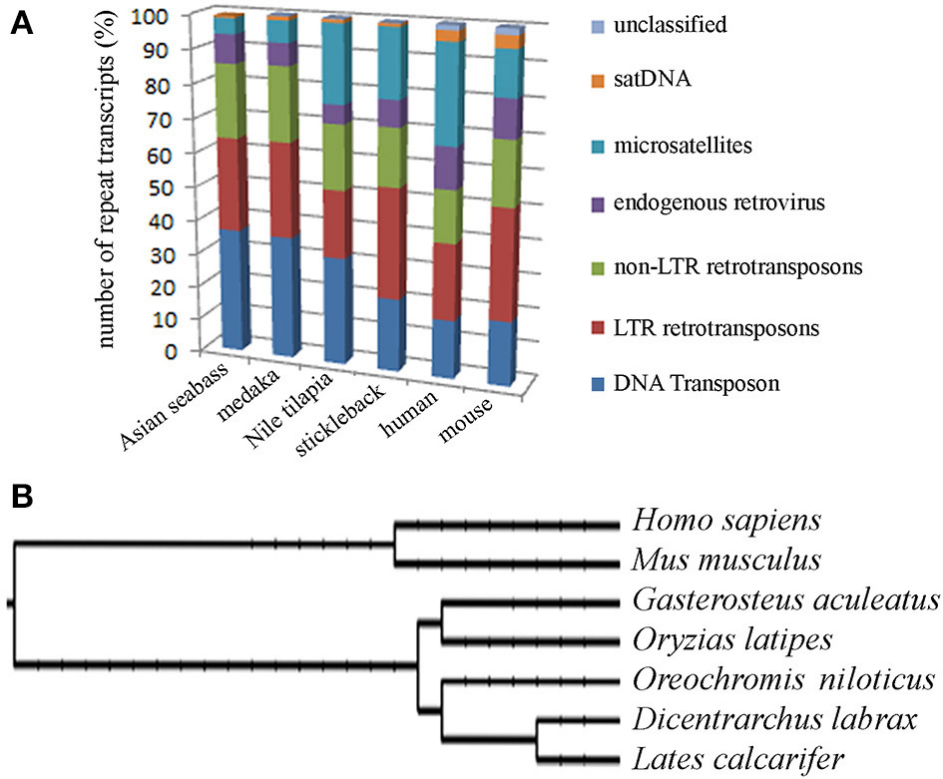
**Teleost and mammalian transcriptomes show similarity to the non-coding regions of genomes as they also contain various amounts of different dispersed and tandem elements. (A)** Comparative analysis of the relative proportion of repeat types in the transcriptome of four teleosts and two mammals. The repeat content of the Asian seabass transcriptome is closer to that of the Japanese medaka than those of the Nile tilapia and the three-spined stickleback. On the Y axis, the percentage of repeat transcript sequences with Repbase hits from all the transcripts are shown. **(B)** Phylogenetic relations between human (*Homo sapiens*), mouse (*Mus musculus*), three-spined stickleback (*Gasterosteus aculeatus*), medaka (*Oryzias latipes*), Nile tilapia (*Oreochromis niloticus*), European seabass (*Dicentrarchus labrax*), Asian seabass (*Lates calcarifer*) were generated by Tree of Life interactive tool (Letunic and Bork, 2011).

The second approach was based on the analysis of the draft assembly of Asian seabass transcriptome that contained a high proportion of unknown transcripts (57%). Their comparison with Repbase showed only a 10% overlap. In total, 14,283 various repeat-containing transcripts were found, their size ranged from 26 to 3600 bp (Supplementary Tables S3, S4). They could be divided into seven classes of repeats: DNA transposons, LTR retrotransposons, non-LTR retrotransposons, endogenous retroviruses, microsatellites, satellites and unclassified (Supplementary Tables S3–S5). When the relative proportion of repeats was compared among four teleost (Asian seabass, Japanese medaka, Nile tilapia and three-spined stickleback) and two mammalian (mouse and human) species, the repeat profile for the Asian seabass was the closest to that of the Japanese medaka (Figure 3A, Supplementary Table S6). While the overall percentage of repeats in the six vertebrate genomes was similar, the teleost transcriptomes contained a lower percentage of repetitive elements than the mammalian ones (Table 2).

**Table 2:**
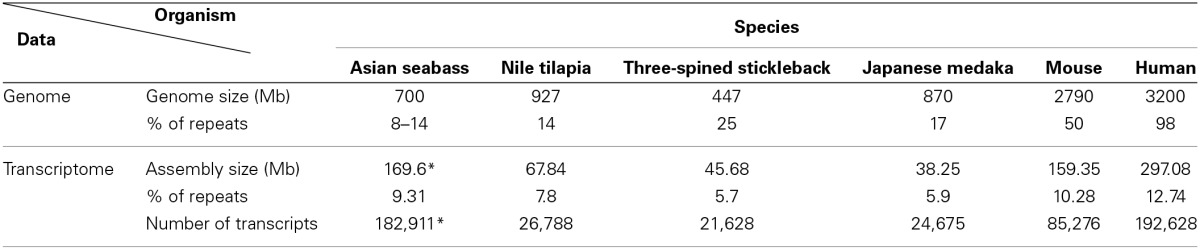
**Comparative analysis of the repeat content of teleost and mammalian transcriptomes and genomes showed distinct differences between groups and among species**.

^*^Incomplete assembly, in progress.

Data for analyses—with the exception of those from Asian seabass—were obtained from NCBI (www.ncbi.nih.gov) and Ensembl (www.ensembl.org) databases.

Sequences that showed homology with characterized satDNA-like repeat elements from other fish genomes were selected from the draft transcriptome assembly (Supplementary Tables S3, S5). One of them aligned with a mosaic satellite from the zebrafish genome (MoSat_DR; GenBank ID: DP000237), while two other fragments showed sequence homology with two different sex chromosome-specific DNAs: the first with repetitive AT-rich DNA sequences from the Y chromosome of the Mediterranean fruit fly, *Ceratitis capitata* (YREP_CC; GenBank ID: AF115330; Supplementary Table S1) and the second with W-specific satellite DNA (GGSat; GenBank ID: X57344, Supplementary Table S1) from the chicken genome (Kawai et al., 2007). The three repeats were cloned and sequenced (Table 1); their sequences showed more than 70% of homology with the corresponding reference sequences listed above. Southern-blot hybridization of gDNA with MoSat_SB and distribution of PCR fragments yielded satellite-like ladder patterns with about 100 bp increments (Figures 2B,C). On the other hand, the PCR distribution fragments for YRep_SB and GGSat_SB were dispersed (i.e., didn't produce the expected ladder) (Figure 2C).

**Figure 4:**
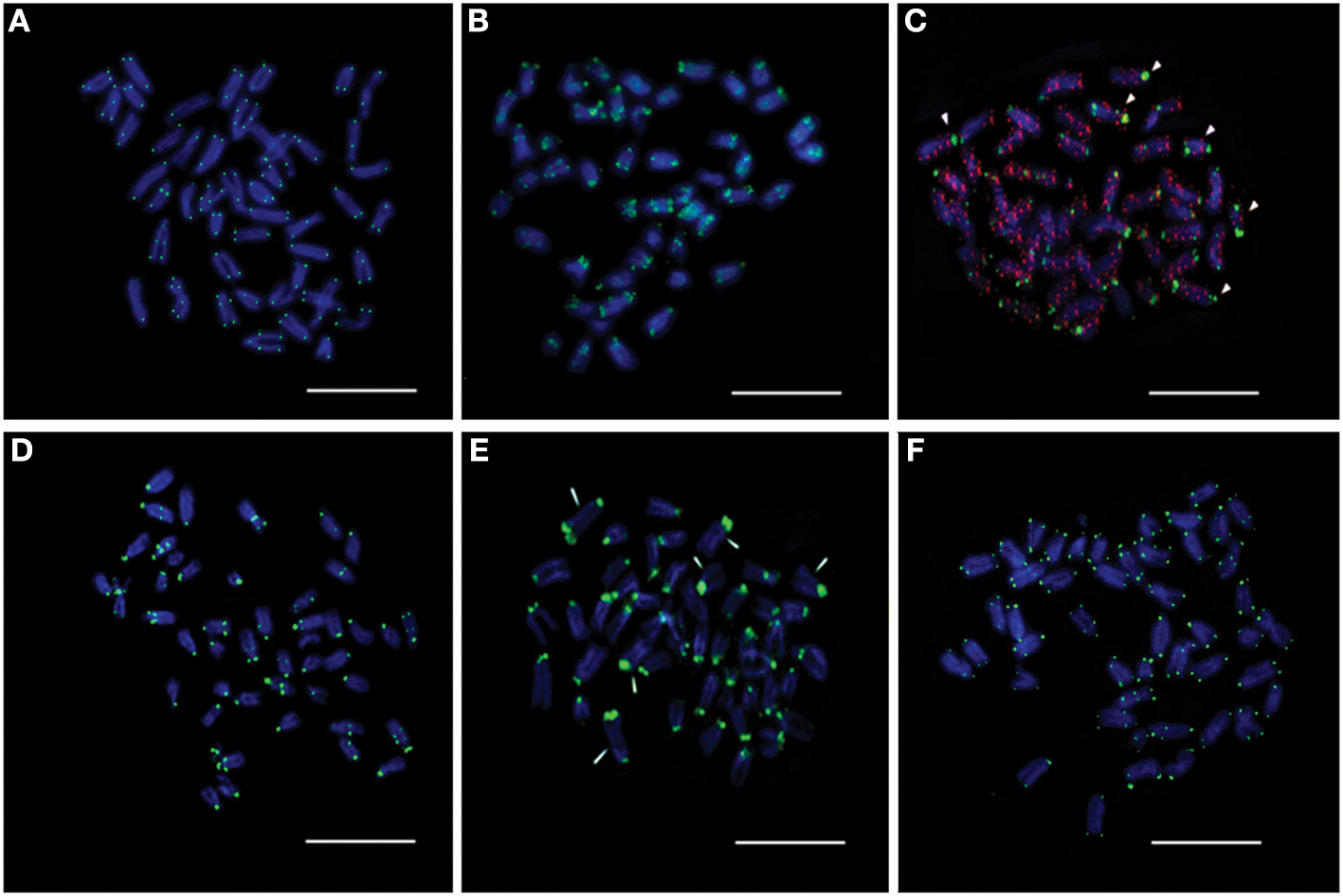
**The chromosomal distribution of seven cloned Asian seabass repeats determined by FISH assay. (A)** Rex1_SB; **(B)** MAUI_SB; **(C)** YRep_SB (green) and GGSat_SB (red); **(D)** OnSat_SB; **(E)** MoSat_SB, and **(F)** Telomere. Chromosomes were counterstained with DAPI (blue). Arrowheads indicate chromosomes with enriched signals. Bar—5 μm.

Among the retrotransposon elements, the Rex group is the most highly represented in the different fish species. We managed to clone two Rex1-related sequences, Rex1_SB and MAUI_SB, from the Asian seabass using specific primers (Table 1). Next, we determined the chromosomal localization of these cloned repetitive sequences. Cytogenetic mapping of these CR1 non-LTR retrotransposons showed stronger signals at the periCEN and telomeric (Tel) regions of all chromosomes and weaker ones on the chromosome arms (Figures 4A,B). The FISH signals of YREP_SB were seen at periCEN regions of all chromosomes and accumulated at 3 pairs of acrocentric chromosomes in the pretelomeric (preTel) and periCEN regions (Figure 4C), whereas those of GGSAT_SB were dispersed through all the chromosomes (Figure 4C). SatDNAs occupied the periCEN regions of all chromosomes (Figures 4D,E). OnSat_SB and MoSat_SB FISH signals were both enriched mostly at the periCEN regions of all chromosomes (Figure 4D), but the latter were also detected in the preTel and periCEN regions of three pairs of acrocentric chromosomes (Figure 4E). All teleosts analyzed so far showed the presence of a standard telomeric repeat (TTAGGG)n (Chew et al., 2002). In the Asian seabass, telomere signals were detected only at the ends of the chromosomes (Figure 4F).

### At least 8% of the asian seabass genome contains repeats and some of them show differential expression in the gonads

The relative amount of OnSat_SB, MoSat_SB, YREP_SB, GGSat_SB, Rex1_SB, and MAUI_SB sequences in the Asian seabass genome was estimated using dot-blot hybridization (Figure 2D). The results showed that around 4–10% of the genome contained CR1-type non-LTR retrotransposons, MAUI_SB and REX1_SB, whereas about 2% consisted of two types of satDNA: OnSat_SB and MoSat_SB (Figure 2D). Together, the six repeat types constituted approximately 8–14% of the Asian seabass genome.

Seven repeats belonging to tandem and dispersed classes of repetitive DNA have been discovered. All of them identified matching sequence reads in the transcriptome, indicating that they produce RNA products (Supplementary Table S3) and four of them (MoSat_SB, Rex1_SB, YRep_SB, and GGSat_SB) were subjected for quantitative analysis of expression levels. While the relative expression level of MoSat_SB and Rex1_SB in the testis was higher than the ovary (*P* < 0.05; pairwise fixed reallocation randomization test), those of YRep_SB and GGSat_SB did not exhibit such differences between the two gonads (Table 3). In the brain, the expression level of Rex1_SB, but not MoSat_SB, also showed an increase compared to that of the ovary.

**Table 3:** **Comparison of the levels (Ct) of repeat-derived transcripts from the gonad and brain of five male and five female adult Asian seabass individuals detected differential expression of MoSat_SB and Rex1_SB between the testis and ovary**.

**Repeat/Gene name**	**Ct value**		
	**Ovary**	**Testis**	**Brain**
MoSat_SB	20.40 ± 1	26.42 ± 1.58^*^	23.6 ± 1.18
Rex1_SB	23.92 ± 1.24	28.80 ± 1.72^*^	28.23 ± 1.4^*^
YRep_SB	19.60 ± 0.98	20.72 ± 1.24	19.37 ± 0.98
GGSat_SB	19.60 ± 0.89	22.48 ± 1.34	18.7 ± 0.87
Elongation factor 1-alpha	34.00 ± 1.7	35.40 ± 2.1	32.00 ± 1.92
Ribosomal protein L8	17.50 ± 0.87	16.80 ± 1	16.00 ± 0.8

* Indicates the Ct values with statistical difference. Statistically significant differences were calculated by the pairwise fixed reallocation randomization test (^*^P < 0.05). The Ct values of male and female brain were similar, thus the data were combined. Elongation factor 1-alpha and ribosomal protein L8 were used as reference genes.

### The genomic organization of two ribosomal DNAs showed species-specific and evolutionarily conserved features

**Figure 5:**
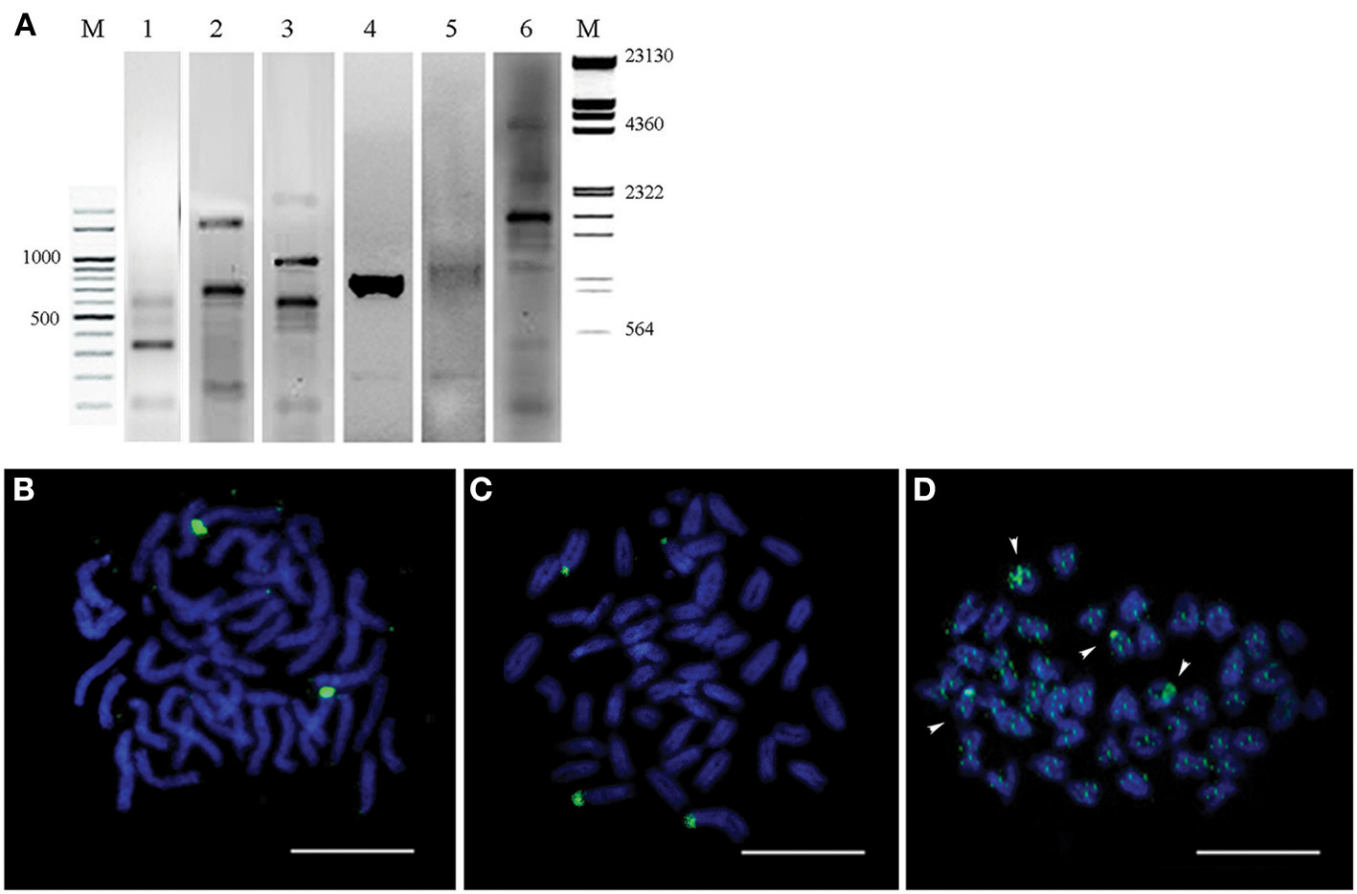
**Genomic hybridization and chromosomal location of two ribosomal DNAs (5S and 18s rDNA). (A)** 18S rDNA (Lanes 1, 2) and 5S rDNA (Line 3) PCR products of the *Lates calcarifer* genome visualized on 2% agarose gel. Southern blot hybridization of the Asian seabass total DNA digested by restriction endonucleases HinfI and HindIII with the DIG-labeled 18S rDNA (Line 4) and 5S rDNA (Line 5) probes. 18S rDNA PCR product from A6 and N6 BACs was used as a DIG-labeled probe for Southern blot hybridization of the same two BAC clones digested by HinfI (Line 6). M—molecular mass markers. The corresponding molecular masses are indicated on the left and right of the figure. **(B)** Fluorescent *in situ* hybridization onto metaphase chromosomes with 5S rDNA probe. **(C)** Fluorescent *in situ* hybridization onto metaphase chromosomes with18S rDNA probe. **(D)** Chromosomal distribution of the A6 and N6 BACs. Arrows indicate chromosomes with enriched signals. Bar—5 μm.

Evolutionarily conserved fragments of 5S rDNA and 18S rDNA (Table 1) were obtained through PCR amplification with teleost-specific primers. Two fragments of 18S rDNA 306 bp and 756 bp as well as 556 bp fragments of 5S rDNA (120 bp of these sequences are conserved across the species analyzed) were cloned and sequenced (Figure 5A, Lanes 1–3).

Labeled probes were produced for both rDNA types and they were hybridized onto the chromosomes. FISH signals for the two rDNAs appeared on separate chromosomes: the signal for 5S rDNA was located on a single chromosome pair (Figure 5B), whereas that for 18S rDNA was found in the periCEN region of two pairs of chromosomes (Figure 5C).

The 18S rDNA-specific primers were used for screening a BAC library to identify clones containing this ribosomal DNA. Two BAC clones (A6&N6), which partially overlapped forming a contig of ca. 101 kb length were chosen. According to the Southern data, the contig was expected to contain short fragments of 18S rDNA (Figure 5A, Lanes 4–5). When the two BAC clones forming the contig were labeled and used as probes for hybridization, the signal was chromosome-specific: it showed accumulation in the periCEN regions of two acrocentric chromosomes, and two periTel regions of two small acrocentric chromosomes, while a slightly dispersed signal was observed on all chromosome arms (Figure 5D).

**Figure 6:**
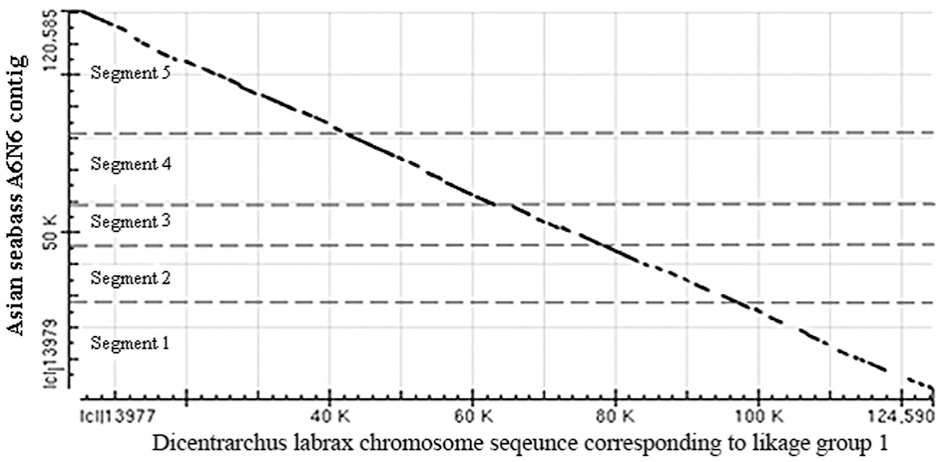
**Dot-matrix analysis of the arrangement of five contigs in the A6N6 scaffold showed good alignment with sequences corresponding to LG1 of European seabass**. Vertical—18S rDNA scaffold, horizontal—European seabass (*Dicentrarchus labrax*) chromosome sequence corresponding to linkage group 1; (GenBank: FQ310506.3; 13,066,894–13,196,483 bp). For the identity of the five contigs see Supplementary Table S2. These two species are distantly located on the phylogenetic tree of teleosts, but belong to the same order (see Figure 3B).

The two BAC clones were sequenced using the HiSeq paired-end protocols and assembled into contigs. Five non-overlapping contigs were produced, their combined length was 120,780 bp (Supplementary Table S2), whereas the length of the A6N6 contig formed by the two BAC clones selected for sequencing was 101 kb according the physical map (FPC contig 2979; Xia et al., 2010). Then single-end reads produced by the Ion Torrent together with the HiSeq data from the five contigs were used for scaffolding based on: (1) alignment with known fish genomic sequences; and (2) the coverage for each contig by the Ion Torrent reads. The total length of the resulting A6N6 scaffold was 120 kb, which was longer than the combined length of the two BAC inserts. A dot-matrix analysis of the resulting A6N6 scaffold was performed with chromosome sequences corresponding to Linkage group 1 of the European seabass (*Dicentrarchus labrax*; GenBank: FQ310506.3); the results showed 92% of identity between the two sequences (Figure 6). The A6N6 scaffold did not produce any annotated BLASTN hits. In absence of LG-specific markers in these sequences, we were unable to match A6N6 to a unique chromosome(s). The reference sequence from European seabass genome contains incomplete fragments of the rDNA. Comparison to these indicated that our scaffold contained incomplete, short fragments of 18S rDNA (the polyA tail and 40–80 bp). Masking with Repbase sequences showed that only 6.9% of the scaffold yielded hits with annotated repeats. All repeat fragments found were generally short (14–314 bp; Supplementary Table S7). Around 14% of the scaffold produced hits when compared with BLAST against the Asian seabass transcriptome database.

## Discussion

Detailed information about repetitive elements present in a particular genome can provide useful information that can be used to improve de novo genome assemblies. For this reason, we have initiated a repeat inventory of the Asian seabass, as part of our genome project. The transcriptome and genome of this teleost species were searched for repetitive elements with experimental and bioinformatic tools. Studies involving comparative genomics have revealed that most vertebrate lineages contain different populations of dispersed elements, including retrotransposons and DNA transposons, and significant differences could be observed in their proportions among species of the same lineage. Highly repetitive satellite sequences and moderately repetitive transposable elements form constitutive heterochromatin, which is mostly located in the preTEL, CEN and periCEN regions of chromosomes (for reviews see Choo, 1997; Kawai et al., 2007; Enukashvily and Ponomartsev, 2013). Our data have demonstrated that transcriptomes show similarity to the non-coding regions of genomes as they also contain various dispersed and tandem elements in different teleost species (Figure 3A; Supplementary Tables S3–S6). The current size of the Asian seabass transcriptome assembly (169.6 Mb) is much larger than those of the other fish species (38–68 Mb; Table 1), presumably due to the following reasons: (i) it is partial and yet to be mapped onto the genome; (ii) while the other teleost transcriptomes were mostly generated with the aid of Sanger- and/or pyrosequencing of cDNAs, the Asian seabass transcriptome was produced by high throughput next generation sequencing resulting in a much larger amount of sequence data; (iii) Ensembl transcripts were obtained from RNA-seq-based gene models (Flicek et al., 2012), whereas the current version of the Asian seabass transcriptome wasn't; and (iv) there is a possibility of “inflation” due to unique transcripts that have retained introns (i.e., sequencing of immature mRNAs). Although our “in progress” Asian seabass transcriptome is derived from over a dozen different adult tissues and samples collected at several developmental stages, we cannot exclude the possibility that we have missed repeat-derived transcripts that were active, but not expressed in the samples used for sequencing.

**Figure 7:**
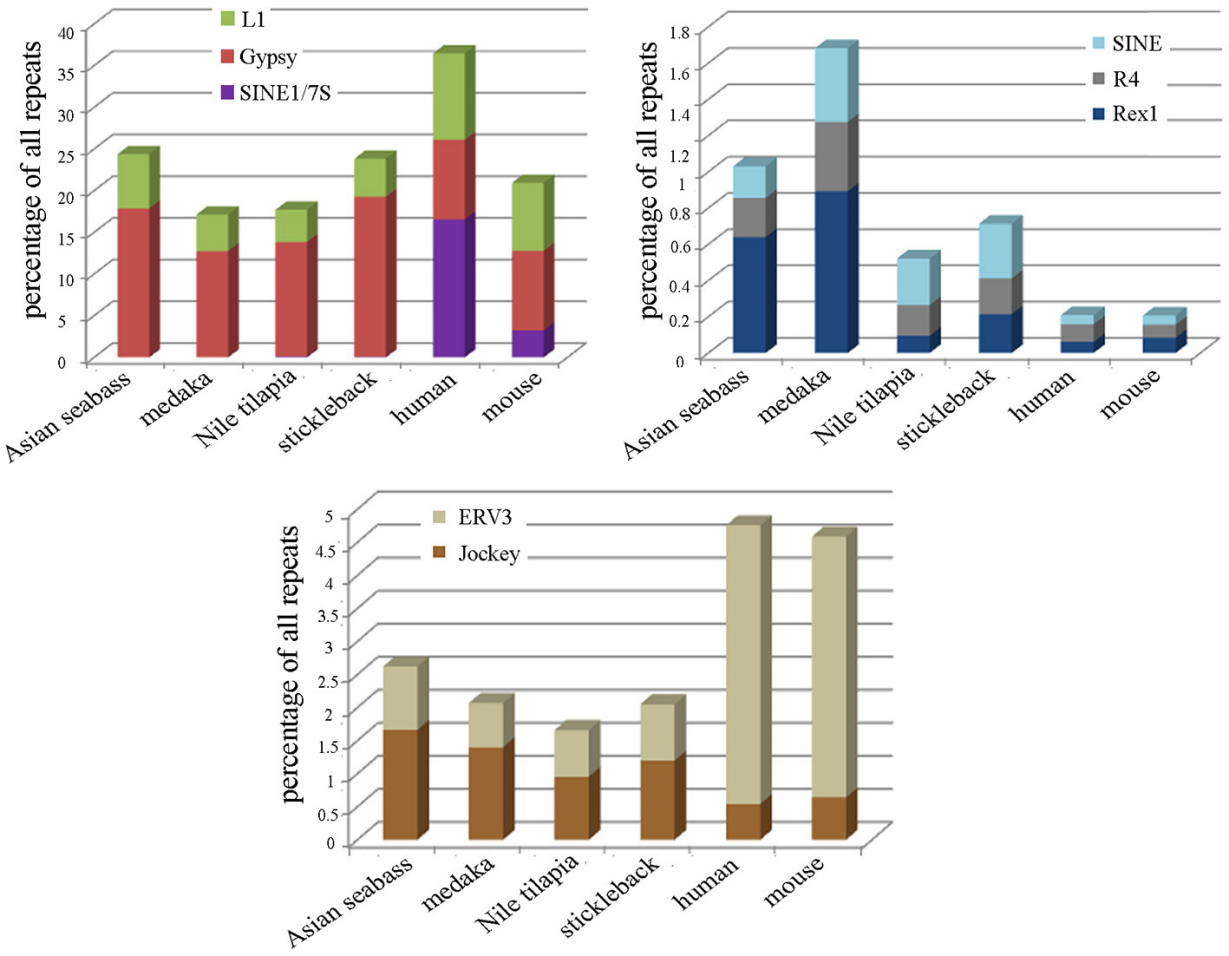
**The relative proportion of eight repeats is different among the transcriptomes of teleosts and mammals**. The following repeats were analyzed: Jockey, ERV3, Rex1, R4, SINE, SINE/7SL, Gypsy, and L1 in the transcriptomes of Asian seabass, Japanese medaka, Nile tilapia, three-spined stickleback, human and mouse. Proportions are shown as the percentage of all repeat-containing transcripts per species (Y-axis). The size of transcriptome for the species is indicated in Table 2.

Repeat-derived transcript sequences form only ca. 6–13% of the Asian seabass transcriptome, whereas most of the teleost genomes assembled so far contain more than 14% of various repeat types (Table 2). This indicates that the large differences in the repeat content between species are due to the repeat DNAs being present not only in the non-coding regions of genomes (Kramerov and Vassetzky, 2005), but also in the transcriptomes (Table 2, Supplementary Tables S3–S6). Studies involving comparative genomics have revealed that most vertebrate lineages contain different populations of retrotransposable elements and DNA transposons with significant differences frequently observed among species of the same lineage (Ferreira et al., 2011). The repeat-derived transcripts of teleosts are generally short, their average length is 50–400 bp; the largest transcript length is no longer than 3.6 kb for Asian seabass compared to 6 kb for human transcriptome (Supplementary Table S3). The longest repeat-derived transcripts in human and mouse generally belong to the non-LTR retrotransposon L1, ERV, or Mariner/TC1 groups (Supplementary Table S3), while those in fish transcriptomes are part of a partially overlapping set of repeat types, excluding ERV. The longest repeat transcript for Asian seabass is MAUI_SB with 3575 bp (Supplementary Table S3). Some dispersed, transcript-derived repeats from retrotransposons, such as MAUI (Poulter et al., 1999), Gypsy, Rex1 (Volff et al., 2000, 2003), Bell, and TART (Casacuberta and Pardue, 2003) are longer than 1 kb and show similarity with each other across different fish genomes (Supplementary Table S3). The maintenance of *D. melanogaster* telomeric DNA is accomplished by repeated retrotransposition of the non-LTR retrotransposon HeT-A/TART specifically to telomeres, in contrast to the maintenance of tandem arrays of species-specific simple DNA repeats at telomeres by telomerase in many eukaryotic organisms (Maxwell, 2004). The result of transcript analysis was consistent with TART producing an array of heterogeneous sense and antisense transcripts (Maxwell, 2004). TART, a non-LTR retrotransposon, was also found in the Asian seabass transcriptome (Supplementary Tables S3, S6). This repeat belongs to the Jockey group (Casacuberta and Pardue, 2003) and its transcript size is longer in teleosts than in mammals (Figure 7). The DNA transposon-based transcript content in human and mouse is lower than those of fishes, but that of the endogenous retrovirus group is higher in mammals (Figure 3). We demonstrated a fish-specific transcriptome-based repeat profile with a few group of repeats, where the proportion of groups Rex1, SINE, R4, Gypsy, and Jockey is higher in fishes then in human and mouse (Figure 7), while that of ERV3 and L1 is lower (Figure 7; Supplementary Table S6). The relative amount of primate-specific SINE1/7SL or Alu transcripts is about three magnitudes higher in human than in fishes. The same tendency has been observed in the distribution of R4/Rex6, Rex1, and Gypsy retrotransposons based on comparative analysis of whole genome shotgun sequences of pufferfishes (Japanese fugu and green-spotted pufferfish), zebrafish, human and mouse genomes (Volff et al., 2003; Shirak et al., 2009). The short sequence length of most repeats from different fish genomes and transcriptomes, including satDNA (Volff et al., 2003; Shirak et al., 2009), indicates that a large part of fish-specific repeat sequences are not characterized.

Moreover, although transcriptomes tested so far tend to have a unique satDNA distribution profile, all investigated fish transcriptomes have similar sets of satellite DNA (Supplementary Table S5). For example, the major satDNA transcript in Asian seabass is OnSat_SB, which has shown similarity with Nile tilapia genome, and is present in the other fish transcriptome datasets, albeit with lower level of similarity. On the other hand, the two main satDNA sequences of the Nile tilapia genome, Satellite A (SATA) and Satellite B (SATB) (Shirak et al., 2009), have not been found in the partial transcriptome data of Asian seabass so far (Supplementary Table S5). The phylogeny based on mitochondrial and nuclear markers suggests independent amplification from the “library.” The existence of a “library of satDNA” has been previously demonstrated experimentally (Mestrović et al., 1998; Bruvo et al., 2003; Pons et al., 2004) and suggested a common ancestor whose genome harbored all or most of the major satDNA families present in the living species at low copy numbers (Pons et al., 2004). Thus, we can confirm that satDNA is shared by a group of related organisms at variable copy number (Mravinac and Plohl, 2010). The existence of short RNA fragments originating from satDNA has been shown experimentally (Rizzi et al., 2004; Valgardsdottir et al., 2005; Enukashvily et al., 2009; Ting et al., 2011; Kuznetzova et al., 2012). Sequencing of such transcripts confirmed that they consisted of satellites only. They are polyadenylated (Rizzi et al., 2004; Enukashvily et al., 2009) and exhibit mostly intranuclear spot-like localization (Valgardsdottir et al., 2005; Kuznetzova et al., 2012). The reported length of satDNA transcripts in mammals varied from 20 bp to 5 kb (Valgardsdottir et al., 2005; Ting et al., 2011) probably because the transcripts were revealed at different stages of their processing. In the various cell types and at different stages of development or cell cycle, the transcription of satellites is asymmetrical: either the sense or the antisense strand is transcribed (Rizzi et al., 2004; Valgardsdottir et al., 2005; Enukashvily et al., 2009; Ting et al., 2011; Kuznetzova et al., 2012). Our experiments have demonstrated a different expression level of satDNA (MoSat_SB) and Cr1 non-LTR retrotransposon Rex1_SB, among the ovary and testis—but not between the male and female brain—of adult Asian seabass (Table 3).

The distribution pattern of constitutive heterochromatin is a good chromosome marker for some teleosts (Kantek et al., 2009; Vicari et al., 2010). Different strategies have been utilized for isolating repetitive DNA sequences: (1) traditional method genomic DNA restriction (Beridze, 1986); (2) re-association kinetics based on *C0t*–*1* DNA (Devine et al., 1997; Langmead et al., 2009); (3) the microdissection of chromosomes submitted to C-banding and subsequent amplification of heterochromatic sequences using DOP-PCR or WGS amplification primers (Cioffi et al., 2013); and (4) bioinformatic analysis of WGS and NGS sequences (Komissarov et al., 2011) and transcriptome data (Jiang et al., 2012). Some of these approaches have been used to investigate the repeats in this study.

**Figure 8:**
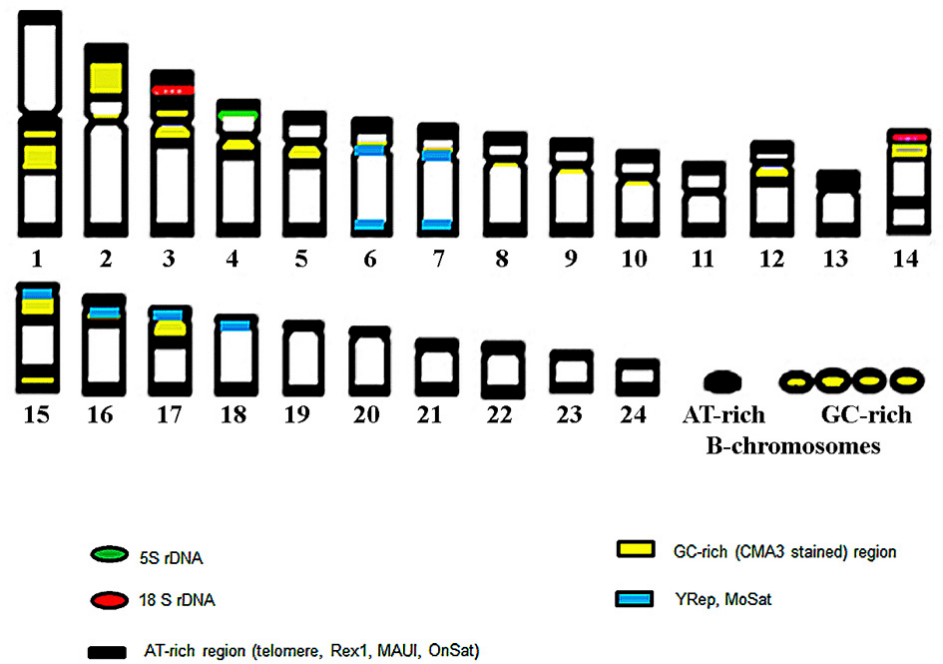
**The ideogram of the chromosome complement of Asian seabass exhibiting cytogenetic mapping of ribosomal sequences, repeats and, heterochromatic, AT- and GC-rich regions**. Altogether, eight of the 24 autosomal chromosome pairs showed a unique hybridization profile with the various probes.

Six new repeats belonging to tandem and dispersed groups of repetitive DNA have been identified, they constitute ~8–14% of the Asian seabass genome. The MoSat_SB and YRep_SB repeats are enriched on individual chromosomes, while the other four—Rex1_SB, MAUI_SB, OnSat_SB, and GGSat_SB—are generally distributed on the autosomes throughout the karyotype (Figures 4, 8). The analysis of the chromosomal location of repeated elements demonstrated that they are, in most cases, compartmentalized in heterochromatic regions and the periCEN regions of chromosomes tend to show a unique repeat distribution as has been observed in several other vertebrates (Beridze, 1986; Enukashvily and Ponomartsev, 2013). Our results have demonstrated that the compartmentalization of repeat elements is mostly restricted to heterochromatic AT-rich segments, whereas most of the chromosomes have large GC-rich blocks within their centromeric regions (Figures 1, 8). The AT-rich B-chromosomes of the Asian seabass contain chromatin which differs from heterochromatin identified in periCEN regions of the autosomal chromosomes (Figure 8). The GC-rich B-chromosomes, as chromosomes with potentially higher recombination rate could be useful for future characterization *Lates* populations throughout the Indian, South-East Asian and Australian regions.

Transposable elements (TEs) can be organized in clusters or dispersed throughout the genome. CR1 elements regulate gene activity and located within genes, as MAUI from the fugu genome (Poulter et al., 1999). Among the CR1 clade of LINE element family of non-LTR retrotransposons, Rex was characterized for the first time in the genome of the *Xiphophorus* and it was found to be widely present in different fish genomes (Volff et al., 2000). Phylogenetic analysis revealed that Rex1 retrotransposons were frequently active during fish evolution. They formed multiple ancient lineages, which underwent several independent and recent bursts of retrotransposition and invaded fish genomes with variable success rate (Volff et al., 2000; Ferreira et al., 2011). The divergence between two cloned sequences of Rex1 in the Asian seabass was ~10%, at the same time about 20% of divergence was found among Rex1 elements isolated from three different teleost species (Volff et al., 2000, 2003). The physical mapping of different Rex elements showed that they were primarily compartmentalized in the periCEN heterochromatic regions, although dispersed or clustered signals in euchromatic regions were also observed (Figure 4A, Valente et al., 2011). The presence of TEs in heterochromatin can be correlated with their role in structure and organization of heterochromatic areas (such as centromeres) or with the lower selective pressure that act on these gene-poor regions. Rex elements were also concentrated in the largest chromosome pair of the Nile tilapia, *Oreochromis niloticus*. This chromosome pair is supposed to have originated by fusions, demonstrating the possible involvement of TEs with chromosome rearrangements (Valente et al., 2011). Rex1, 3, and 6 are non-LTR elements that have been active during the evolution of different fish lineages. In the family of *Cichlidae* (*Perciformes*), Rex elements are organized in clusters within the genome of the majority of the species (Martins et al., 2004). Rex elements showed a wide distribution among fishes and could be observed both in the periCEN region and euchromatic regions of chromosomes. However, at present, due to the lack of an assembled genome, we are unable to resolve their exact location in relation to the periCEN region. Rex elements can also be associated with genes involved with sex determination (Volff et al., 2000). In this study, we have demonstrated the existence of Rex1-derived transcripts in Asian seabass; moreover, their expression levels were significantly lower in the adult ovary than in the testis (Table 3).

The Nucleolus Organizer Regions (NORs) are highly repetitive genome sites related to rRNA synthesis (Preuss and Pikaard, 2007; Pinhal et al., 2008). NORs present small, active transcription sites (evolutionally conserved 120 bp for 5S rDNA and 300–2800 bp for 18S rDNA) and highly variable non-transcribed spacing segments with their own structural dynamics, in which the presence of transposons located close to the genome regions has been identified (Martins et al., 2004). rDNA sequences and their chromosomal location have proven to be valuable as genetic markers to distinguish closely related species and also in the understanding of the dynamic of repetitive sequences in the genomes. In this study we have shown that the chromosomal position of 5S rDNA and 18S rDNA probes in Asian seabass is similar to those observed in different fish species (Martins et al., 2004; Mantovani et al., 2005; Cioffi et al., 2010; Merlo et al., 2010; Nakajima et al., 2012). At the cytogenetic level, the 5S rDNA probes hybridized mostly near to the centromere region; while the 18S rRNA gene probe has identified variable positions across different fish species (Martins et al., 2004; Mantovani et al., 2005; Cioffi et al., 2010; Merlo et al., 2010; Nakajima et al., 2012).

In order to improve our understanding of their organization, rDNA sequences were analyzed in the Asian seabass genome. Two clones were identified through hybridization with 18SrDNA-derived probes from a BAC library; they formed a single contig (A6N6). They were sequenced with two NGS platforms (Illumina HiSeq and Ion Torrent) and assembled. The A6N6 contig is extremely rich in repeats, containing conserved (Supplementary Table S7) and novel sequences. Some of them are chromosome-specific, whereas others are dispersed throughout the chromosomes (Figure 5D). Due to the shortness of reads, the assembled repeats tend to be highly fragmented. In teleosts, the spacer portion of the major rDNA is generally GC-rich (Schmid and Guttenbach, 1988), whereas our A6N6 scaffold shows a high (59%) AT content. It was shown in the red wolf fish (*Erythrinus erythrinus*) that rDNA sequences were co-localized with the Rex3 retrotransposable element in the centromeric heterochromatin (Valente et al., 2011) and they have also shown strong association with Tc1/Mariner and Rex elements (Schmid and Guttenbach, 1988; Cioffi et al., 2010; Merlo et al., 2010; Nakajima et al., 2012). The presence of Tc1/Mariner and Rex1_SB repeats was observed in the A6N6 scaffold (Supplementary Table S7). Transcripts derived from the A6N6 scaffold showed sequence homology with a predicted gene (PDZ domain containing ring finger 3) from fish and amphibian genomes. The synteny of Asian seabass A6N6 scaffold with a European seabass linkage group (Figure 6) and their phylogenetic association (Figure 3B) strengthened the idea that the chromosomes have undergone rearrangements during evolution. These rearrangements were likely mediated by retrotransposon activity in which the insertion of the retrotransposable element into rDNA sequences created an rDNA-transposon complex that moved and dispersed in the karyotype (Cioffi et al., 2010; Nakajima et al., 2012).

The transcriptome and genome of Asian seabass were searched for repetitive elements with experimental and bioinformatics tools. In summary, our data indicate that the sequence and structure of most Asian seabass repeat DNAs are likely to be unknown. Detailed analysis of the completed genome assembly will provide the final proof for this suggestion. The approach used for the analysis of repeats in this study has also yielded useful knowledge only about evolutionarily conserved repeats. All analyzed sequences, except Tel, belong to the periCEN part of chromosomes (Figure 8), they could form chromosome-specific periCEN patterns of compartmentalization and the transcripts of some show differential expression between the adult gonads of Asian seabass sex.

### Conflict of interest statement

The authors declare that the research was conducted in the absence of any commercial or financial relationships that could be construed as a potential conflict of interest.
