# Supplementary figures and images for: Primary analysis of repeat elements of the Asian seabass (Lates calcarifer) transcriptome and genome
Source: Front Genet. 2014 Jul 25;5:223. doi: 10.3389/fgene.2014.00223 (PMC4110674; doi:10.3389/fgene.2014.00223)

## The main steps of repeat inventory. Sequencing, assembly and analysis.

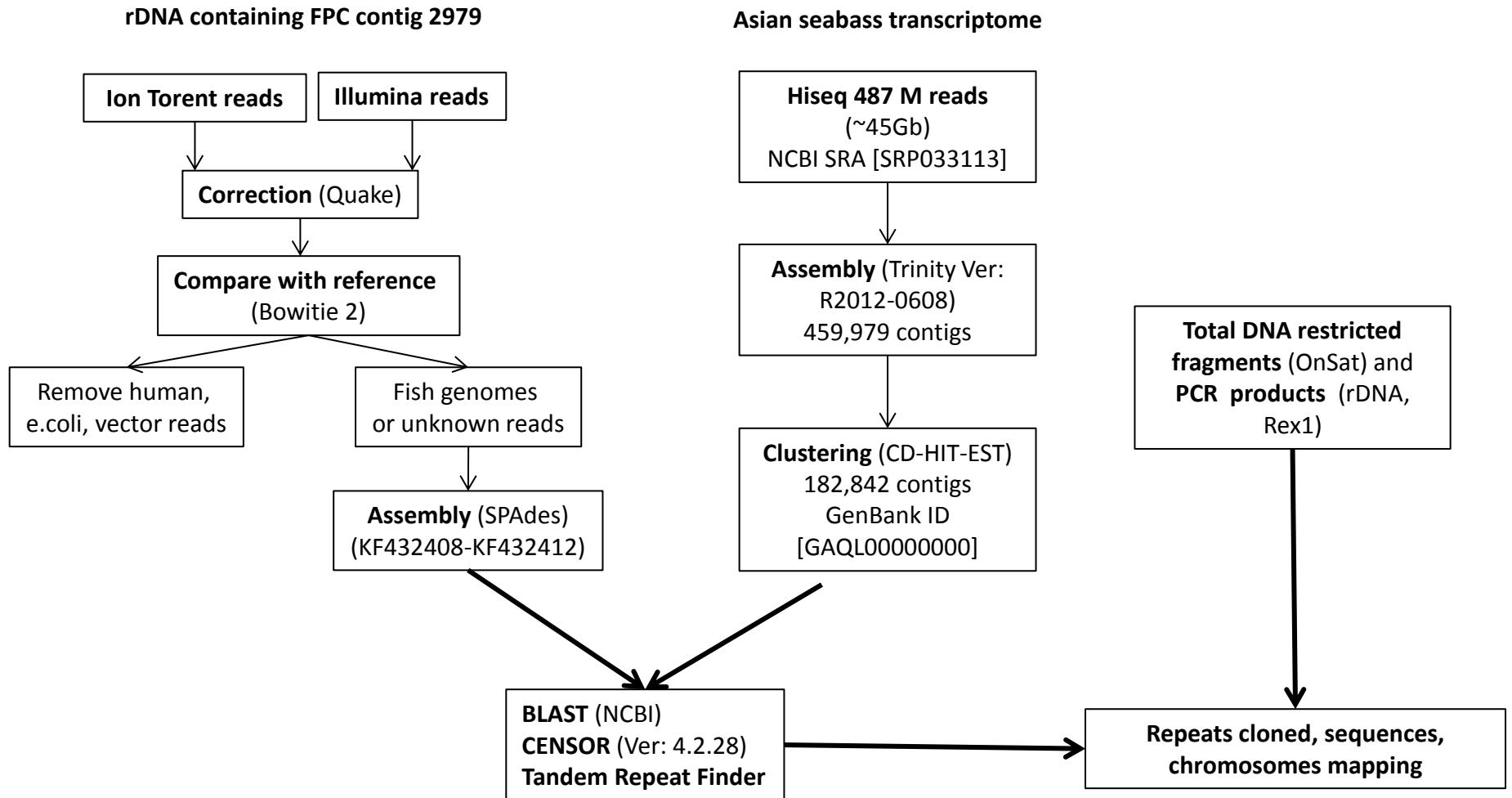

Supplement: Supplementary file 1 [file Presentation1.PDF]
