# Supplementary material for: Primary analysis of repeat elements of the Asian seabass (Lates calcarifer) transcriptome and genome
Source: Front Genet. 2014 Jul 25;5:223. doi: 10.3389/fgene.2014.00223 (PMC4110674; doi:10.3389/fgene.2014.00223)
Supplement: Supplementary file 2 [file DataSheet1.ZIP › 82745_Kuznetsova_Table_1.DOCX]

**Supplementary Table S1:** Primer sequences used for PCR-amplification of repeats

| Sequence name | Primer sequences (forward/reverse) | Reference sequences, NCBI or Repbase ID |
| --- | --- | --- |
| 5S rDNA (including the NTS region) | A 5’-TAC GCCCGA TCT CGT CCG ATC-3’  B 5’-CG GCT GGTATG GCC GTA AGC-3’ | 5S rDNA with 120 bp conserved part ([Martins et al. 2004](#_ENREF_36)) |
| 18S rDNA | 18Sf 5’- CCG CTTTGG TGA CTC TTG AT-3’  18Sr 5’-CCG AGG ACCTCA CTA AAC CA-3’  18S-1 5'-TCA AGA ACG AAA GTC GGA GG-3’  18S-2 5'-GGA CAT CTA AGG GCA TCA CA-3’ | 18S rDNA ([Mantovani et al. 2005](#_ENREF_34)) |
| Rex1 | RTX1-F1 5'-TTCTCCAGTGCCTTCAACACC-3’  RTX1-R3 5'-CCCTCAGCAGAAAGAGTCTGCTC-3’ | Rex1; JX576302-JX576350 ([Poulter et al. 1999](#_ENREF_44)) |
| YREP_CC | CcYf 5’-GCTCGAAGACATGCATTGAA-3’  CcYr 5’-GACGGTAAGTGCCATTCGTT-3’ | AF115330 |
| GGSAT | GGSATR 5’-CCTCCACGAAAATACCGCCT-3’  GGSATF 5’-AAACTTGTGTTTTCGGCGGG-3’ | X57344 ([Kawai et al. 2007](#_ENREF_24)) |
| MoSat_DR | MoSatF 5’-TCACAAGCCAGCCTCAAAGT-3’  MoSatR 5’-GACAGTTGCTAGGGTGCAGT-3’ | DP000237, Zebrafish mosaic satellite repeat |
| ONSATB | Sat2R 5’-ATGGCTCCAAGAGACTTTTTTGGT-3’  Sat2F 5’-CGTCTGCAAATTTTGGTAAGTTTT-3’ | S57288 ([Franck and Wright 1993](#_ENREF_19)) |
