# Supplementary material for: Primary analysis of repeat elements of the Asian seabass (Lates calcarifer) transcriptome and genome
Source: Front Genet. 2014 Jul 25;5:223. doi: 10.3389/fgene.2014.00223 (PMC4110674; doi:10.3389/fgene.2014.00223)
Supplement: Supplementary file 2 [file DataSheet1.ZIP › 82745_Kuznetsova_Table_2.DOCX]

**Supplementary Table S2:** The five contigs produced by sequencing the two BAC clones (A6 & N6) using the HiSeq paired-end protocols and assembling them the resulting reads.

| Contig/NCBI accession number | Length (bp) | Coverage  (-fold) |
| --- | --- | --- |
| Segment1/KF432408 | 28,206 | 2209.21 |
| Segment 2/ KF432409 | 17,536 | 2041.32 |
| Segment 3/ KF432410 | 13,293 | 2196.92 |
| Segment 4/ KF432411 | 22,590 | 2334.98 |
| Segment 5/ KF432412 | 39,408 | 1839.5 |
